# Molecular characteristics of novel immune subtypes of HCC based on lncRNAs related to immune disorders

**DOI:** 10.1038/s41598-022-13013-7

**Published:** 2022-05-26

**Authors:** Qiyao Zhang, Xiao Yu, Shuijun Zhang, Wenzhi Guo, Yuting He

**Affiliations:** 1 Department of Hepatobiliary and Pancreatic Surgery, The First Affiliated Hospital of Zhengzhou University, Zhengzhou, 450052 China; 2 Key Laboratory of Hepatobiliary and Pancreatic Surgery and Digestive Organ Transplantation of Henan Province, The First Affiliated Hospital of Zhengzhou University, Zhengzhou, 450052 China; 3 Open and Key Laboratory of Hepatobiliary and Pancreatic Surgery and Digestive Organ Transplantation at Henan Universities, Zhengzhou, 450052 China; 4 Henan Key Laboratory of Digestive Organ Transplantation, Zhengzhou, 450052 China

**Keywords:** Gastrointestinal cancer, Oncology

## Abstract

As one of the most malignant cancers and despite various treatment breakthroughs, the prognosis of hepatocellular carcinoma (HCC) remains unsatisfactory. The immune status of the tumor microenvironment (TME) relates closely to HCC progression; however, the mechanism of immune cell infiltration in the TME remains unclear. In this study, we performed a new combination algorithm on lncRNA expression profile data from the TCGA-LIHC cohort to identify lncRNAs related to immune disorders. We identified 20 immune disorder-related lncRNAs and clustered HCC samples based on these lncRNAs. We identified four clusters with differences in immune cell infiltration and immune checkpoint gene expression. We further analyzed differences between groups 1 and 3 and found that the poor prognosis of group 3 may be due to specific and non-specific immunosuppression of the TME, upregulation of immune checkpoint pathways, and activation of tumor proliferation and migration pathways in group 3. We also developed a prognostic model and verified that it has good stability, effectiveness, and prognostic power. This study provides a basis for further exploration of the immune cell infiltration mechanism in HCC, differential HCC prognosis, and improvement of the efficacy of ICIs for the treatment of HCC.

## Introduction

As the fifth most malignant tumor, hepatocellular carcinoma (HCC) causes more than 800,000 deaths worldwide every year^1,2^. Although exciting breakthroughs have been made in surgical treatment, radiotherapy, and targeted therapy over the past few decades, available treatment strategies are still limited due to the lack of early symptoms and high recurrence rates in HCC patients^3–5^. Recently, researchers have focused on identifying new biomarkers for early diagnosis and new treatment strategies, including immunotherapy^6–8^. Immune checkpoint inhibitors (ICIs) have shown therapeutic effects and few side effects against a variety of malignant tumors, including HCC, lung cancer, and pancreatic cancer^9–11^. However, only a small percentage of patients are sensitive to immunotherapy, and the specific mechanism of immunotherapy remains unclear^11,12^. Therefore, exploring the differential responses of HCC to immunotherapy and the underlying molecular mechanisms are important to identify suitable patients for immunotherapy and to improve HCC prognosis.

Recent studies have shown that immune cell infiltration of the solid tumor microenvironment contributes to the evolution and progression of tumors and associates with a variety of clinical indicators^13,14^. The tumor immune microenvironment has been shown to be involved in specific and non-specific immune responses to tumors and in tumor immune escape^15,16^. Long non-coding RNAs (lncRNAs) are non-coding RNAs of ≥ 200 nucleotides, and they have been shown to be involved in biological and cellular functions^17^. lncRNAs have been shown to regulate tumor immunity^7,18^ For example, Runqiu Jiang et al. found that lnc-EGFR binds to epidermal growth factor receptor (EGFR) specifically and blocks its interaction with and ubiquitination by c-CBL to induce EGFR expression and promote regulatory T cell (Treg) differentiation, thereby affecting the tumor immunosuppressive state^19^.

However, it is unknown which other lncRNAs affect the tumor immune microenvironment. In this study, which was inspired by single-sample gene set enrichment analysis (ssGSEA), we used lncRNA expression profiles from The Cancer Genome Atlas Liver Hepatocellular Carcinoma (TCGA-LIHC) and biomarkers of 24 immune cell to screen for lncRNAs that associate with immune disorders and to identify new immune subtypes of HCC. We identified 20 immune-related lncRNAs, performed a cluster analysis of HCC samples based on expression of these lncRNAs, and four cluster groups were identified. We further analyzed groups 1 and 3 and found that the prognosis for group 3 was poor. We determined that the poor prognosis of group 3 may be due to suppression of immune cell infiltration into the tumor microenvironment (TME), upregulation of immune checkpoints, and activation of a variety of tumor-related pathways, indicating that the group 3 subtype may be sensitive to treatment with ICIs. We also developed a prognostic model of HCC with good predictive power that has been verified by independent data sets, has shown effectiveness and stability, and that functions a tool for further exploring the mechanisms of immune microenvironment disorders in HCC and their relationship to HCC prognosis.

## Results

### Identification of HCC sample immune subtypes based on lncRNA expression

**Figure 1:**
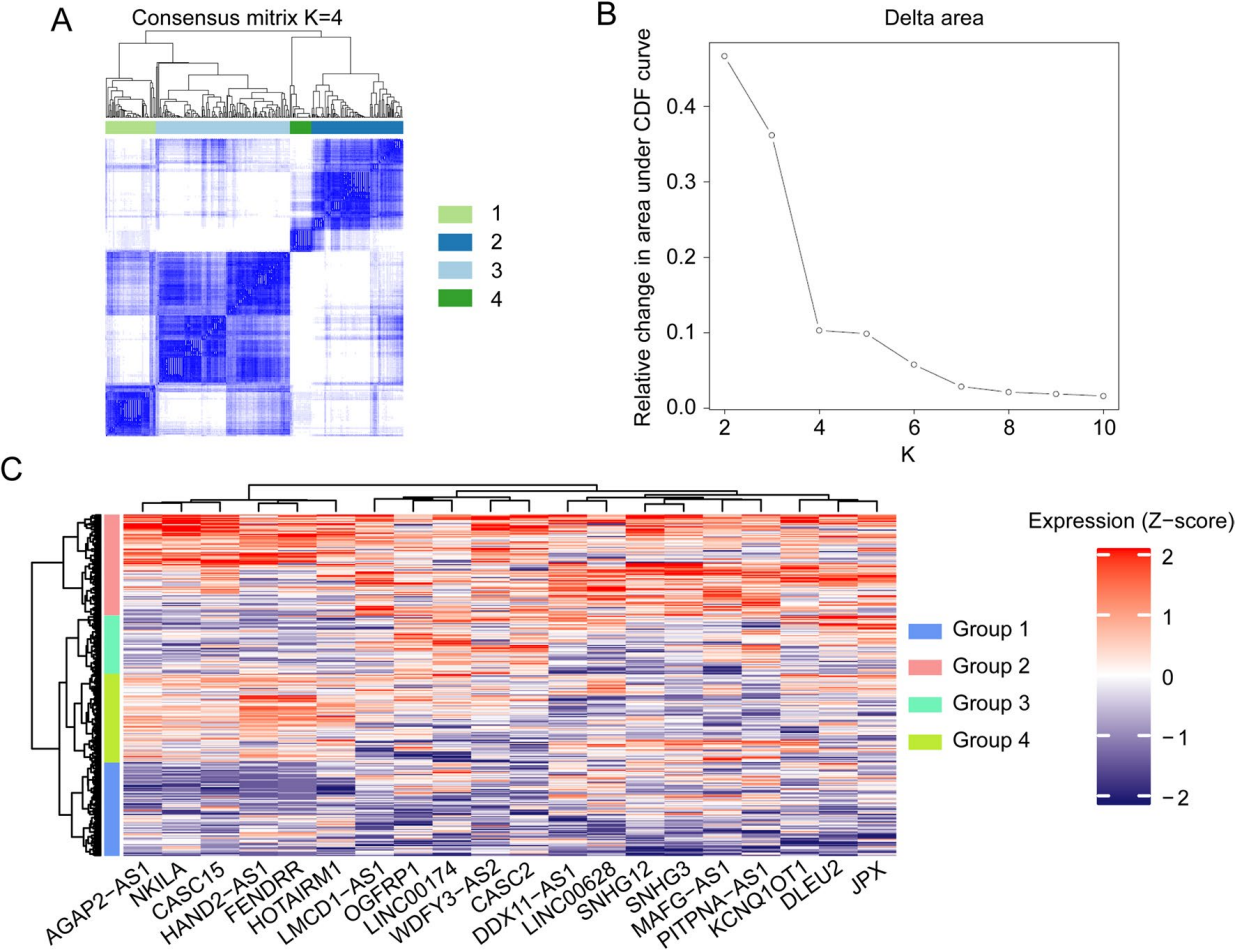
Consensus clustering of HCC samples based on 20 lncRNAs related to immune disorders. (**A**) The consensus clustering map when K = 4. (**B**) K = 4 was considered the optimal number of clusters. (**C**) Heatmap of expression of the 20 immune-related lncRNAs among the different subtypes.

We identified 20 immune-related lncRNAs that may contribute to immune disorders. We clustered HCC samples according to expression of these lncRNAs. Among these 20 lncRNAs, 10 lncRNAs had significant prognostic value, and all of these 10 lncRNAs were risk factors for HCC patients (Supplementary Fig. 1A). The optimal clustering multiple was k = 4 (Fig. 1A–B, Supplementary Fig. 1B). Group 1 contained 87 HCC samples, group 2 contained 136 HCC samples, group 3 contained 45 HCC samples, and group 4 contained 49 HCC samples. The heat map shows that the expression of the 20 immune-related lncRNAs was consistent among groups. In general, the average expression of the lncRNAs is low in group 1 and highest in group 2 (Fig. 1C).

### Immune cell infiltration and prognoses of the immune subtypes

**Figure 2:**
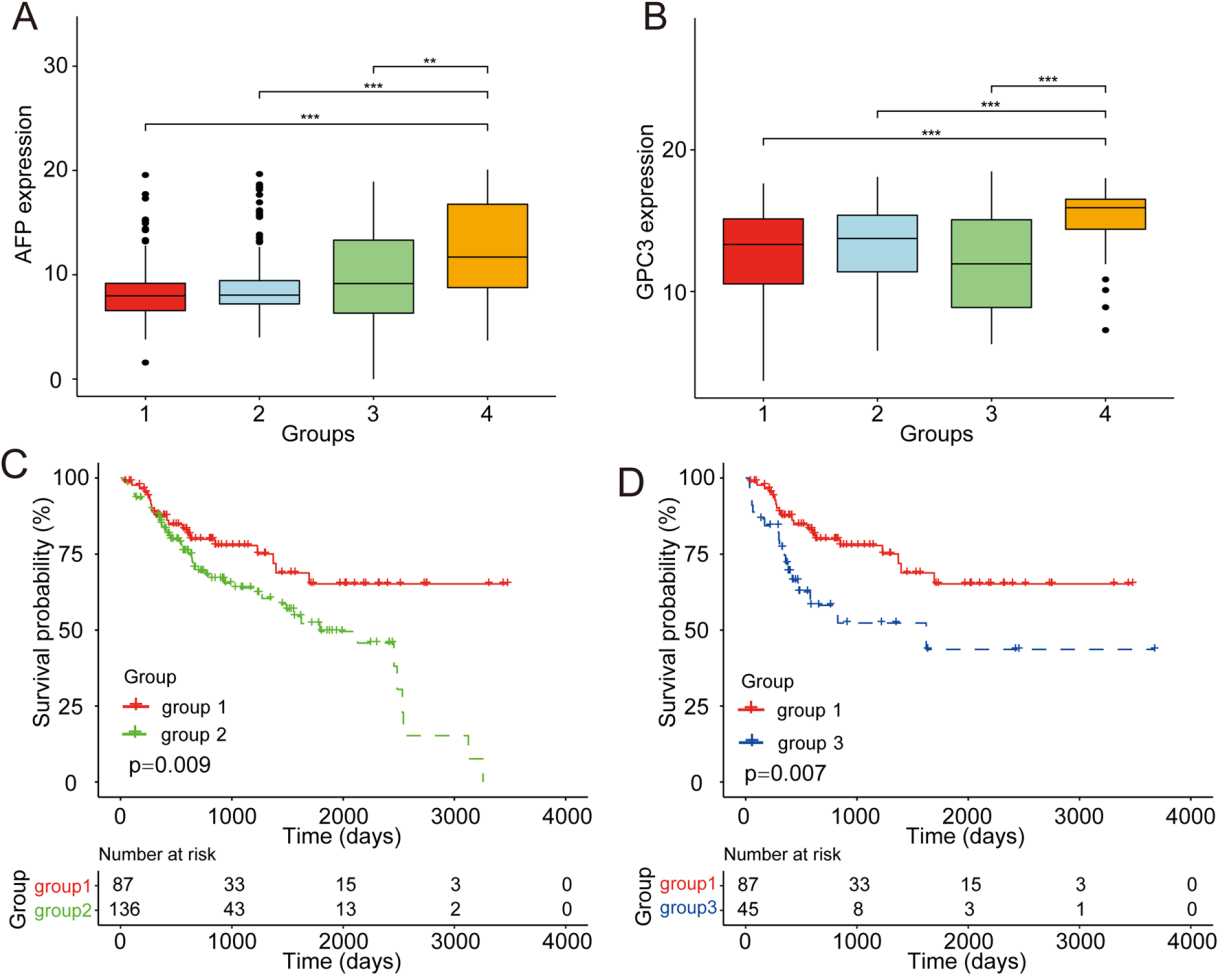
Prognostic differences between subtypes. (**A**) AFP expression differences among subtypes. Groups 3 and 4 had higher AFP expression. (**B**) GPC3 expression differences among subtypes. (**C**) Significantly different prognoses between groups 1 and 2. (**D**) Prognoses with the most significant differences were between groups 1 and 3.

We explored the prognostic differences of the four immune subtype clusters. Expression levels of the HCC specific diagnostic markers alpha-fetoprotein (AFP) and glypican-3 (GPC3) were compared, and significant differences in expression of these two biomarkers were found among the subtype clusters. AFP expression was low in group 1 and high in groups 3 and 4 (Fig. 2A). The highest GPC3 expression was in group 4 (Fig. 2B). Kaplan–Meier (KM) analysis showed significant prognostic differences between groups 1 and 2 and between groups 1 and 3. Compared with group 1, groups 2 and 3 had significantly worse prognoses (Fig. 2C–D). The survival prognoses did not differ between the other groups (Supplementary Fig. 2).

**Figure 3:**
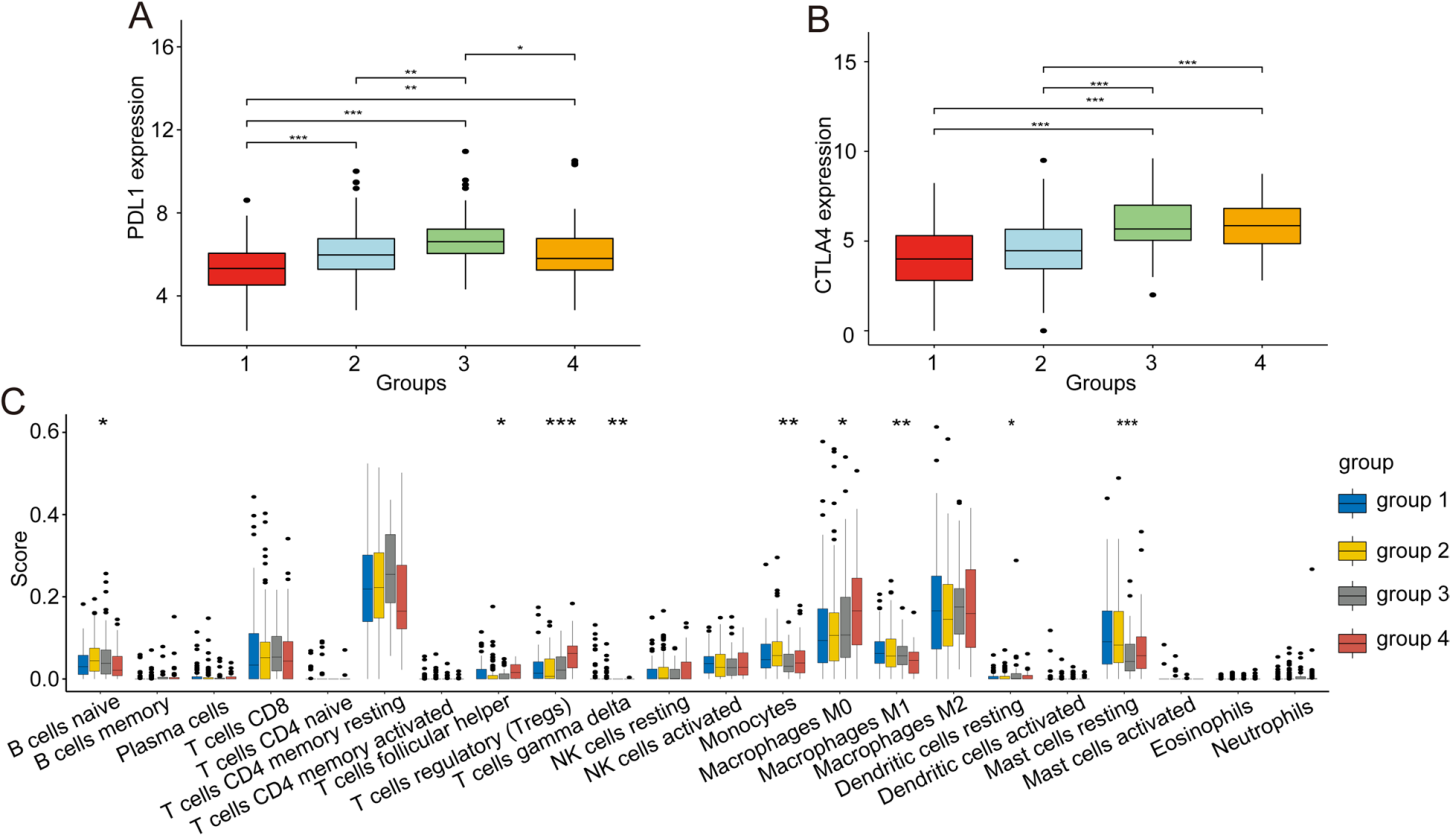
Differences in immune checkpoint gene expression and immune cell infiltration between subtypes. (**A**) Expression of PDL1 differed among the four subtypes, and PDL1 was significantly upregulated in group 3. (**B**) CTLA4 expression differed among subtypes. (**C**) Differences in immune cell infiltration among subtypes was based on ssGSEA, and a certain degree of immunosuppression was found in group 3.

To further characterize the four immune subtypes, we analyzed expression of immune checkpoint genes in the subtypes. The results showed that expression levels of two well-studied immune checkpoint genes, programmed death-ligand 1 (PD-L1) and cytotoxic T-lymphocyte-associated protein 4 (CTLA4), differed significantly among the four subtypes. The lowest PD-L1 and CTLA4 expression levels were in group 1 and the highest PD-L1 and CTLA4 expression levels were in group 3, indicating that the immune system may be "hijacked" via immune checkpoint pathway-dependent methods in group 3 HCC samples (Fig. 3A,B). In addition, to more comprehensively understand the differences in immune checkpoint genes between different groups, we screened multiple immune checkpoint genes from previous studies and compared the expression differences between groups. As shown in Supplementary Fig. 3, almost all immune checkpoint genes were significantly differentially expressed between groups, most were highly expressed in group 3 and low in group 1, which is consistent with our conclusion. We also identified differences in immune cell infiltration among the subtypes. ssGSEA enrichment showed significant differences in infiltration of B cells, helper T cells, Tregs, and mast cells among the subtypes. Interestingly, in group 3, infiltration of monocytes, mast cells, and T follicular helper cells was downregulated, and infiltration of Tregs, which negatively regulate the immune response, was also downregulated (Fig. 3C). CTL, the target cell of immune checkpoint, had the highest degree of infiltration in group 3, although there was no statistical difference. Thus, the lower immune cell infiltration in group 3 may partially explain the poor prognosis of group 3.

### Functional enrichment analysis of the subtypes

**Figure 4:**
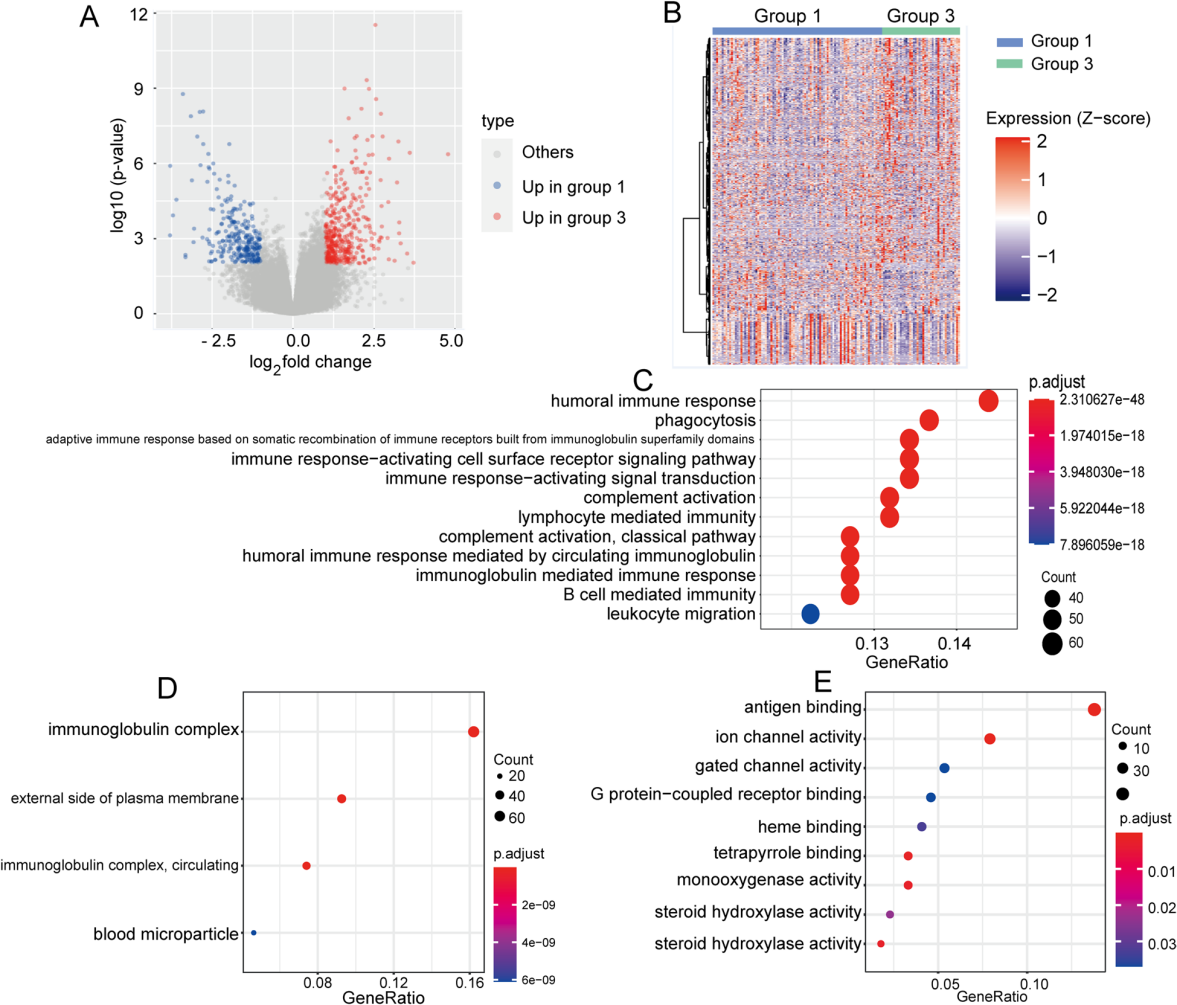
Functional enrichment analysis of groups 1 and 3. (**A**) Volcano map of DEGs between groups 1 and 3. (**B**) Heatmap of DEGs between groups 1 and 3. Most of the DEGs were upregulated in group 3. (**C**) Biological processes (BP) identified from gene ontology (GO) enrichment analyses of DEGs. (**D**) Cellular components (CC) identified from GO enrichment analyses of DEGs. (**E**) Molecular functions (MF) identified from GO enrichment analyses of DEGs. Most enrichments were in specific and non-specific immune response pathways.

Due to the significant difference in prognosis and immune cell infiltration between groups 1 and 3, we chose these two subtypes for further analysis. Differential expression analysis showed significantly different expression of 712 genes between groups 1 and 3 with 282 genes expressed more highly in group 1 and 430 genes expressed more highly in group 3 (Fig. 4A,B, Supplementary Table 5). Functional enrichment analysis showed that almost all of the differentially expressed genes (DEGs) were enriched in immune-related pathways. The enriched biological processes (BP) included the humoral immune response, activation of immune-related cell surface receptors and related pathways, lymphocytes and B cell-mediated immune responses (Fig. 4C). The enriched cellular components (CC) included circulating immunoglobulin complexes and plasma membranes (Fig. 4D). The enriched molecular functions (MF) included activation of ion pathways and binding and activation of antigens and other proteins (Fig. 4E).

**Figure 5:**
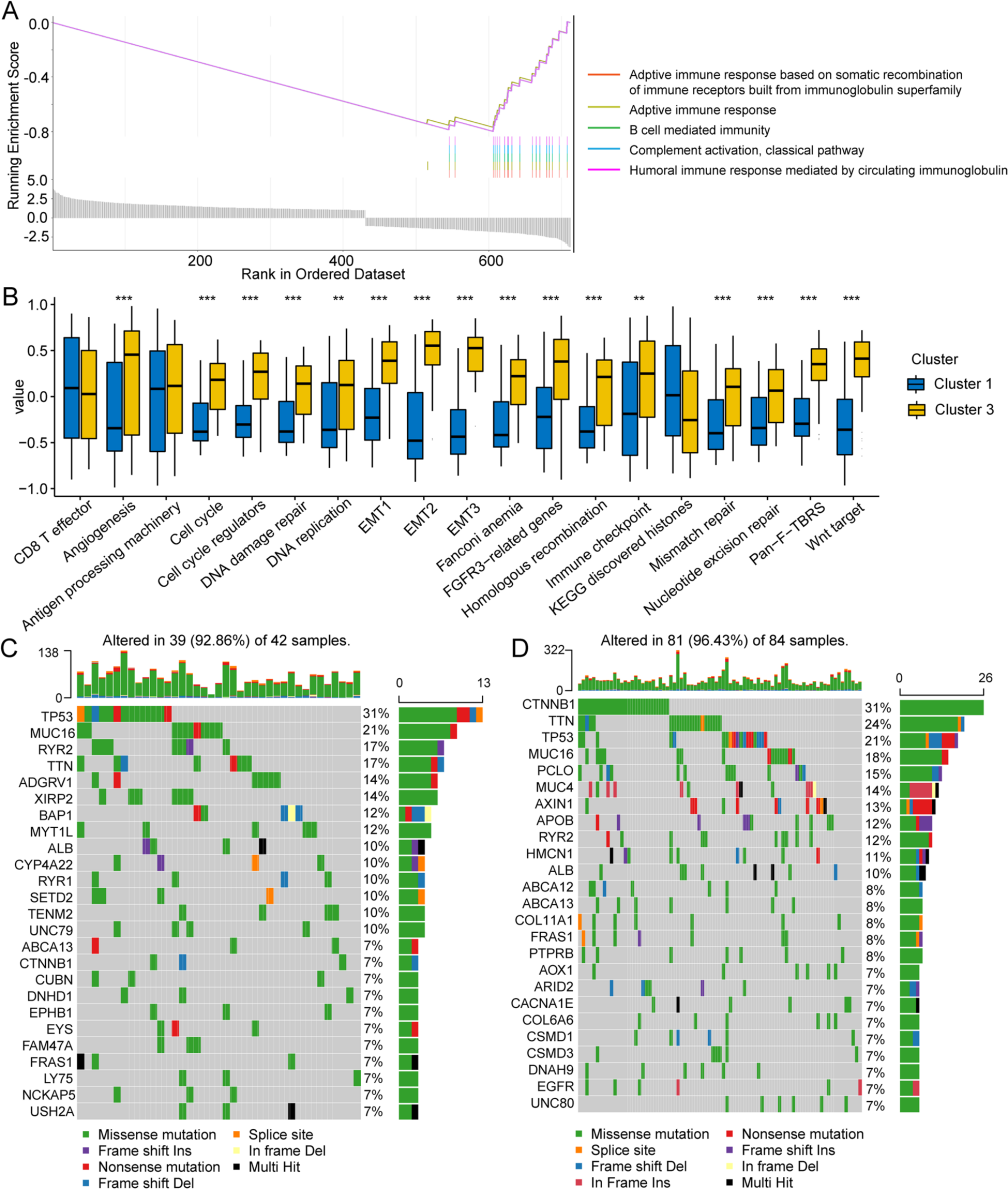
Changes in tumor-related pathways between groups 1 and 3. (**A**) GSEA shows that the immune-related pathways were significantly inhibited in group 3. (**B**) Tumor-related pathways, including tumor proliferation and migration, were activated and immune checkpoints were upregulated in group 3 when compared to group 12. (**C**) A waterfall chart of the 20 genes with the highest mutation frequency in group 1. (**D**) A waterfall chart of the 20 genes with the highest mutation frequency in group 3.

We also performed GSEA on DEGs sorted by foldchange (FC). As expected, a variety of immune-related pathways, especially specific immunity, such as activation of immunoglobulin receptor pathways, B cell-mediated immunity and humoral immunity were enriched in group 1 (Fig. 5A). These data confirmed that suppression of immune cell infiltration in group 3 weakened anti-tumor immunity.

### Tumor-related pathways were activated in group 3

Suppression of the immune response appears partially explains the poor prognosis of group 3. To explore the differences between groups 1 and 3 more comprehensively, we scored the pathways in these two subtypes using the ssGSEA algorithm. The proliferation of tumor cells by cell cycle, DNA damage repair, DNA replication, mismatch repair, homologous recombination, and other pathways, as well as tumor proliferation-related pathways, such as angiogenesis, the epithelial-mesenchymal transition (EMT), and Wnt targets, were activated in group 3 (Fig. 5B). Interestingly, immune checkpoints were significantly upregulated in the group 3 subtype indicating suppression of immune responses through the immune checkpoint pathway in group 3.

In addition, gene mutations between subtypes were analyzed using copy number variation information from the TCGA cohort (Fig. 5C,D). The mutation rate for the CTNNB1 gene, which encodes for β-catenin and has the highest mutation frequency, was highest in group 3 (group 1: 7%, group 3: 31%). β-catenin is an adhesion junction protein, and mutations in β-catenin affect the immune response and could lead to the progression of a variety of tumors, including HCC.

### Establishment of a prognostic model based on DEGs

**Figure 6:**
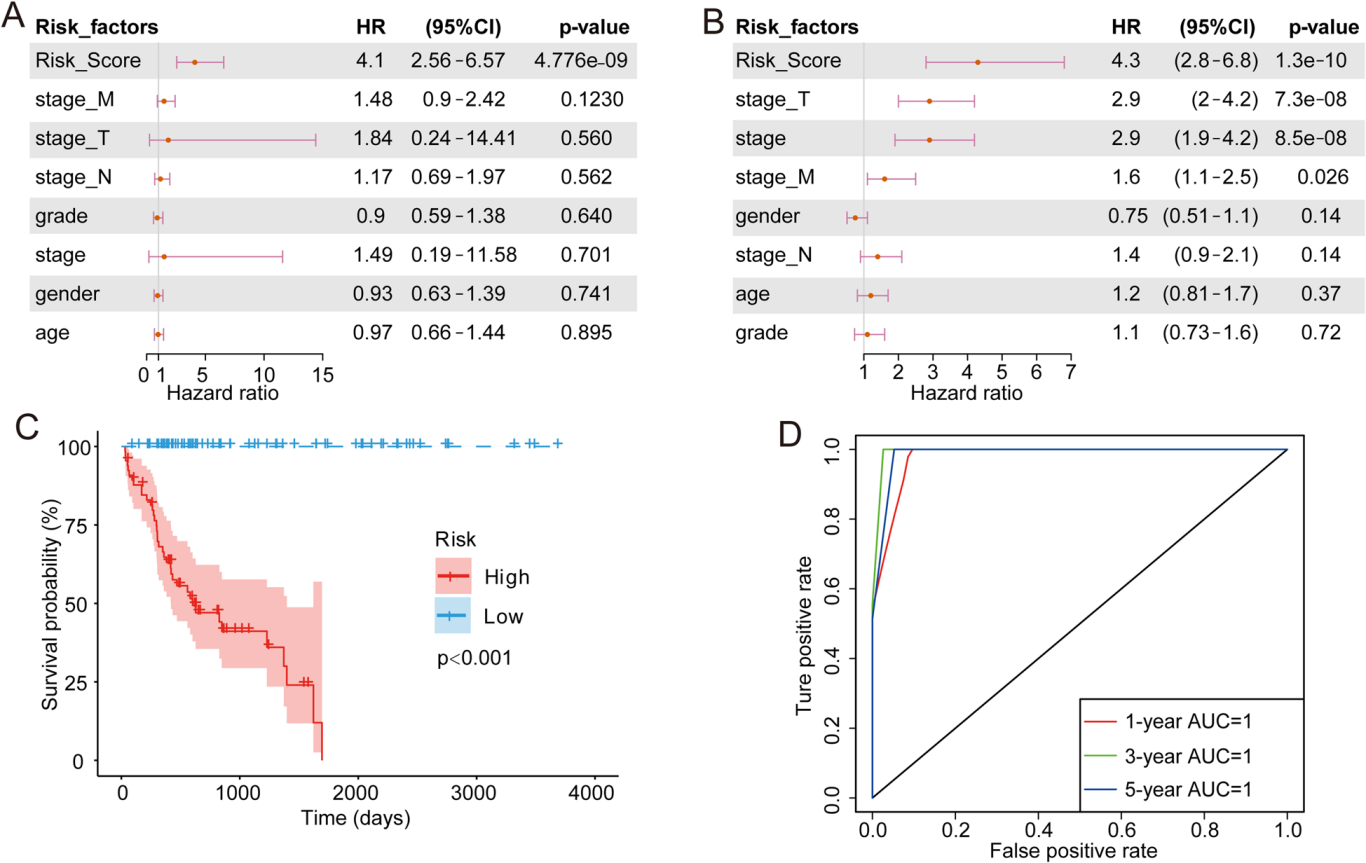
Development of the prognostic model. (**A**) Single-factor cox analysis showed that risk score and stage M had significant impacts on HCC prognosis. (**B**) Multivariate cox analysis showed that, after removing confounding factors, risk score remained a significant independent risk factor. (**C**) High-risk and low-risk groups, which were delineated based on the median risk score, had significant prognostic differences. (**D**) ROC analysis of the prognostic model showed that the model has good prognostic power.

Due to the significant differences in prognosis and molecular characteristics between groups 1 and 3, we performed a single-factor cox analysis of the DEGs. With P < 0.05 as the threshold, 63 genes with significant prognostic value were identified (Supplementary Table 6). Single-factor and multi-factor cox analyses of these 63 genes were performed. The product of the hazard ratio (HR) risk coefficient and the gene expression level was calculated for each gene and then these values were summed to determine the risk score of the sample. Using the median risk score as the threshold, we divide the samples into high-risk and low-risk groups. Single-factor cox analysis showed that only risk score and stage M were significant risk factors for poor prognosis, and risk score had the strongest prognostic value (Fig. 6A). Multivariate cox analysis showed that, after removing confounding factors, risk score remained a significant independent risk factor with an HR of 4.3 (Fig. 6B). Survival analysis showed that the prognosis of the high-risk group differed significantly from the low-risk group; there were no deaths in the low-risk group (Fig. 6C). Receiver operating characteristic (ROC) analysis showed that the area under the curves (AUCs) for 1, 3, and 5 years of the prognostic model reached 1 indicating good prognostic prediction efficiency (Fig. 6D). To further verify the effectiveness of our model, we applied the model to group 2 and group 4, respectively (Supplementary Fig. 4). The results showed that risk score was a significant prognostic factor in both group 2 and group 4. The AUC in group 2 reached 0.83, while those in group 4 were 0.66 (1 year) and 0.59 (3 years). The decline in performance was related to the smaller sample in this group.

### Validation of the robustness of the prognostic model

**Figure 7:**
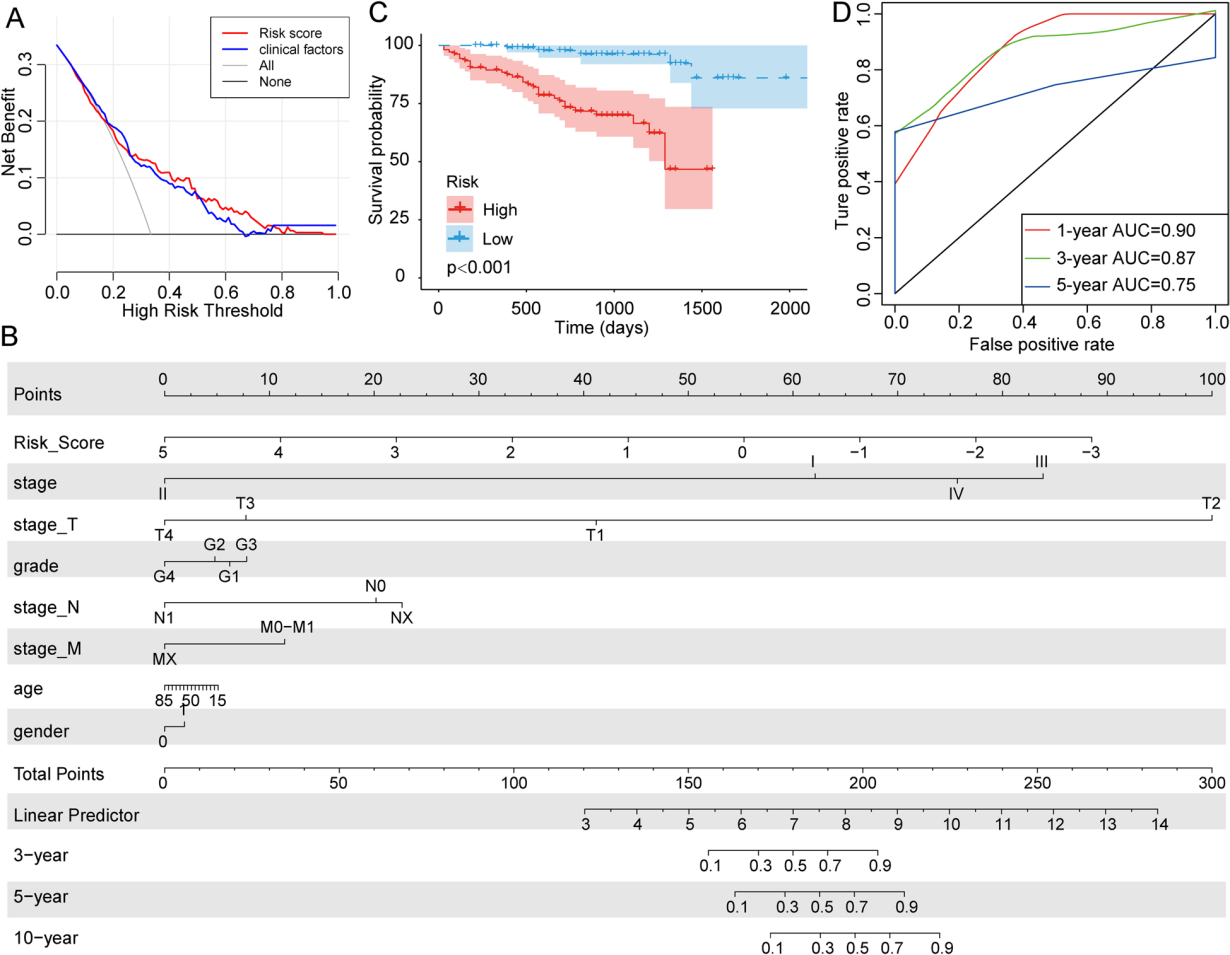
Prognostic model validation and nomogram. (**A**) DCA of the prognostic model showed that, in most cases, decisions based on the risk score had a better net benefit than clinical factors. (**B**) The nomogram showed that risk score had a great impact on prognosis and was relatively stable. (**C**) The prognostic model also showed significant prognostic differences in the ICGC-LIRI-JP cohort. (**D**) The AUCs of this prognostic model with the ICGC-LIRI-JP cohort reached 0.9 (1 year), 0.87 (3 years), and 0.75 (5 years).

Decision curve analysis (DCA) was performed to evaluate the net benefits of different factors for patients. The analysis showed that decisions based on the risk score had positive effects on patient net benefits, and in most cases, had more positive effects than clinical factors (Fig. 7A). The nomogram visually shows the degree of influence of different factors on prognosis using straight lines of unequal length. We used clinical factors, such as risk score, stage, and TNM staging, to develop a nomogram of the prognostic model. The nomogram showed that risk score and stage had the strongest impacts on patient prognosis. In addition, risk score is relatively stable and easy to quantify, therefore it is a good prognostic indicator (Fig. 7B). To verify the effectiveness and stability of the prognostic model, we used the external ICGC-LIRI-JP cohort data set and the same genes and coefficients, and found significant prognostic differences between the high-risk and the low-risk groups in the ICGC-LIRI-JP cohort (Fig. 7C). ROC analysis of this cohort showed that the 1, 3, and 5 year AUCs of this prognostic model were 0.90, 0.87, and 0.75, respectively, which were better than most other prognostic models (Fig. 7D).

## Discussion

It has been shown that a unique tumor immune microenvironment and inhibition of immune cell infiltration leads to escape of tumor cells from immunity and contributes to HCC^21,22^. Tumor cells inhibit the anti-tumor response in the TME through immune checkpoints, such as the PD-1/PD-L1 and CTLA4 pathways^12,23,24^. ICIs can reverse the immunosuppressive state of the TME to a certain extent^25,26^. However, it is puzzling that treatment with ICIs is effective for only a small percentage of patients indicating that the immune cell infiltration status differs significantly among HCC samples. This puzzling phenomenon also indicates that we do not fully understand the characteristics and mechanisms of immune cell infiltration in the TME^27^.

Considering the large number of HCC patients, it is important to evaluate differential immune cell infiltration in the TME for early screening of sensitive populations. In this study, we used the lncRNA expression profile of the TCGA-LIHC cohort to identify lncRNAs with high lncRES scores based on ssGSEA and to identify lncRNAs related to immune cell markers. We then identified 20 lncRNAs that intersected the immune-related lncRNA and immune disorder-related lncRNA categories. Consensus clustering of HCC samples based on these 20 lncRNAs identified four HCC immune subgroups with significant differences. Expression of PD1 and CTLA4 was the lowest in group 1 and the highest in group 3. In addition, ssGSEA showed that group 3 had significant immunosuppressive characteristics. Due to the significant differences in prognosis and immune cell infiltration between groups 1 and 3, we analyzed these groups further. Differential expression analysis showed that the DEGs were expressed highly in group 3. Consistent with our expectations, functional enrichment analysis showed that most of the DEGs were enriched in specific and non-specific immune-related pathways. GSEA also showed inhibition of a variety of immune-related pathways in group 3.

Subsequently, we compared the tumor-related pathway status between subtypes. Surprisingly, pathways related to tumor proliferation and migration, such as angiogenesis, EMT, cell circulation, DNA damage repair, and FGFR3-related genes, were activated in group 3. Interestingly, immune checkpoints were also upregulated in group 3, which is consistent with our previous results. Previous studies have shown that the immune cell infiltration state of tumors relates closely with patient prognosis and that suppression of immunity in the TME promotes uncontrolled proliferation of tumor cells^28,29^. The 20 immune disorders-related lncRNAs that we identified were expressed highly in group 3 and may cause the immunosuppression, the upregulation of immune checkpoint genes, the activation of tumor proliferation and migration pathways, and the poor prognosis of the group 3 subtype. A study by Saima Usman showed that some tumor-related pathways, such as MAPK and EMT signaling pathways, are involved in formation of the immune microenvironment and subject to feedback regulation^30^. Abnormal immunosuppression may promote activation of tumor signaling pathways. Tumor cells may gain additional abilities to escape the immune system by upregulating immune checkpoints^31,32^.

The Wnt/β-Catenin regulatory axis has a key tumor-promoting effect, especially in HCC^33^. More than two-thirds of HCC patients have abnormal Wnt/β-Catenin signaling. Deletion or mutation of CTNNB1, which encodes β-Catenin, are the most common WNT/β-catenin regulatory events in HCC^34,35^. The activation mutation gene that differed the most between groups 1 and 3 was CTNNB1. Mutation waterfall charts showed that the frequency of the CTNNB1 activation mutation was higher in group 3 than in group 1 suggesting abnormal activation of the Wnt/β-Catenin pathway in the group 3 subtype, which is consistent with the upregulation of the Wnt pathway observed in group 3 by ssGSEA.

It has been shown that changes in immune checkpoint gene expression relates closely with the tumor sensitivity to ICIs^36,37^. We identified upregulation of immune checkpoint genes in group 3, and although this upregulation can lead to increased escape of tumor cells from immunity, this subtype may be benefit from immunotherapy. Although we preliminarily analyzed the mechanism of differential HCC prognosis based on newly categorized immune subtypes, the mechanism of formation of the HCC immune microenvironment and the relationship between the immunosuppressive status and activation of tumor-related pathways remain unclear and need to be analyzed in future experiments. Nevertheless, this study suggests that high expression of immune disorder-related lncRNAs causes extensive immunosuppression, activation of tumor-related pathways, and changes in immune checkpoint gene expression; therefore, the lncRNAs identified here may play an important role in tumor progression. Future research on these lncRNAs is required to understand their role in formation of the tumor immune microenvironment and their impacts on HCC prognosis.

## Conclusions

In this study, we identified 20 lncRNAs related to immune disorders and identified new HCC immune subtypes. The differential prognosis of the group 1 and group 3 subtypes may be due to suppression of immune cell infiltration in the TME, the upregulation of immune checkpoints, and activation of a variety of tumor-related pathways in the group 3 subtype. We also developed a prognostic model with good predictive power and stability that can function as a tool to further explore the mechanism underlying immune microenvironment disorders in HCC and to predict HCC prognosis.

## Methods

### Data sources and processing

The TCGA-LIHC and the International Cancer Genome Consortium Liver Cancer-Riken, Japan (ICGC-LIRI-JP) cohorts were downloaded for analysis. We used the TCGA-LIHC cohort as the training cohort (https://portal.gdc.cancer.gov/) and the ICGC-LIRI-JP (https://dcc.icgc.org/) cohort as the verification cohort. RNA expression and clinical data were downloaded on September 1, 2020. RNA-seq data were raw counts. We eliminated genes that were not expressed in the tumor samples, and then log2 converted the expression values of the remaining genes.

### Calculation of rank score for candidate immune-related lncRNAs

For each lncRNA of interest, we ranked coding genes based on correlation of their expression with lncRNA expression. Expression of lncRNA i and gene j across tumor patients was defined as L(i) = (l1, l2, l3, …, li, …, lm) and G(j) = (g1, g2, g3, …, gj, …, gm), respectively. Tumor purity scores across m patients were defined as P = (p1, p2, p3, …, pi, …, pm). We first calculated the partial correlation coefficient (PCC) for expression of lncRNA i and gene j by considering tumor purity as a co-variable:

$$PCC(ij)=\frac{{R}_{LG}-{R}_{LP}*{R}_{GP}}{\sqrt{1-{R}_{LP}^{2}}*\sqrt{1-{R}_{GP}^{2}}}$$

in which RLG, RLP, and RGP are the correlation coefficients for expression of lncRNA i and coding gene j, expression of lncRNA i and tumor purity, and expression of gene j and tumor purity, respectively. In addition, we obtained the P-value for the PCC, which is defined as P(ij). For each lncRNA-gene pair, we calculated the rank score (RS) as follows:

$$RS\left(ij\right)=-log10\left(P\left(ij\right)\right)\times sign(PCC(ij))$$

### Further identification of immune-related lncRNAs based on GSEA algorithm

Genes were ranked based on RS scores and then subjected to enrichment analysis.

Motivated by GSEA, we mapped the genes in each immune-related pathway (Supplementary Table 1) to the ranked gene list and then calculated the enrichment score (ES) based on the GSEA. If there were N genes in the ranked gene list, L = (g1, g2, g3, …, gN), and the ranked score was RS (gj) = rj. We evaluated the fraction of genes in pathway H (“hits”) weighted by their RS and the fraction of genes not in S (“misses”) up to a given position i in L as follows:

$$P\_hit (H,i)=\sum_{\begin{array}{c}{g}_{i}\in H\\ j\le i\end{array}}\frac{{|{r}_{j}|}^{p}}{{N}_{R}}, \mathrm{where} {N}_{R}=\sum_{{g}_{i}\in H}{|{r}_{j}|}^{p}$$

$${P}_{miss}\left(H,i\right)=\sum_{\begin{array}{c}{g}_{j}\notin H\\ j\le i\end{array}}\frac{1}{(N-{N}_{I})}$$

The ES score was the maximum deviation from zero of Phit–Pmiss. In addition, a P value was calculated for each pathway including N_I_ genes as follows:

$$p\left(ES\left(N,{N}_{I}\right)<{ES}_{ik}\right)=\sum_{q=-\infty }^{\infty }{(-1)}^{q}\mathrm{exp}(-2{q}^{2}{ES}_{ik}^{2}n)$$

$$n=\frac{(N-{N}_{I}){N}_{I}}{N}$$

in which ES_ik_ is the ES score between lncRNA i and immune pathway k, N is the number of genes in the ranked list, and N_I_ is the number of genes in the specific immune pathway. P-values were adjusted using the false discovery rate. Moreover, we combined the P-value and the ES score to obtain the lncRES score:

$$lncRES\left(i,k\right)=\left\{\begin{array}{c}1-2p;if ES(ik)>0\\ 2p-1;if ES(ik)<0\end{array}\right.$$

We screened for lncRNAs with lncRES > 0.995 and designated them immune-related lncRNAs. We identified 151 immune-related lncRNAs (Supplementary Table 2).

### Identification of immune-related lncRNAs in HCC by the immune cell abundance identifier (ImmuCellAI) method

We used a previously developed marker gene set of 24 immune cells to evaluate correlations between immune cells and lncRNAs^20^. We used hypergeometric enrichment analysis to identify significant relationships between candidate immune-related lncRNAs and immune cells as follows:

$${P}_{ij}=\frac{{C}_{M}^{K}-{C}_{N-M}^{n-k}}{{C}_{N}^{n}}$$

in which P_ij_ represents the significance of marker gene enrichment between lncRNAi and immune cell j, n represents the number of significantly related mRNAs shared by lncRNAi in HCC samples, M represents the number of marker genes contained in immune cell j, and k represents lncRNAi and immune cell j. K represents the number of significantly correlated marker genes and N represents the number of all marker mRNAs, which is 2025. When k < 3, the test results were not significant. Under the threshold of p < 0.05, we identified significant enrichment relationships between candidate dysregulated immune lncRNAs and immune cells, and we identified 634 immune disorder-related lncRNAs (Supplementary Table 3). Subsequently, we evaluated the intersection of the two types of immune-related lncRNAs we identified above, and identified 20 immune-related lncRNAs in HCC for further analysis (Supplementary Table 4).

### Consensus clustering based on immune-related lncRNAs and CIBERSORT for evaluation of immune cell infiltration subtypes

R package consensus clustering was used for consensus clustering of HCC samples based on 20 immune-related lncRNAs. Differential expression analysis was performed using DESeq2 in the R package. The significance threshold was set to p < 0.01 and |log2FC|> 1. Gene Set Variation Analysis in the R software package was used for functional enrichment analysis. Evaluation of immune cell infiltration by subtype was performed using CIBERSORT.

## Supplementary Information

Supplementary Figure 1.

Supplementary Figure 2.

Supplementary Figure 3.

Supplementary Figure 4.

Supplementary Legends.

Supplementary Table 1.

Supplementary Table 2.

Supplementary Table 3.

Supplementary Table 4.

Supplementary Table 5.

Supplementary Table 6.

Supplementary Legends.

## Data Availability

The original contributions presented in the study are included in the article/supplementary material, further inquiries can be directed to the corresponding authors.
