# Supplementary figures and images for: Molecular characteristics of novel immune subtypes of HCC based on lncRNAs related to immune disorders
Source: Sci Rep. 2022 May 26;12:8905. doi: 10.1038/s41598-022-13013-7 (PMC9135727; doi:10.1038/s41598-022-13013-7)

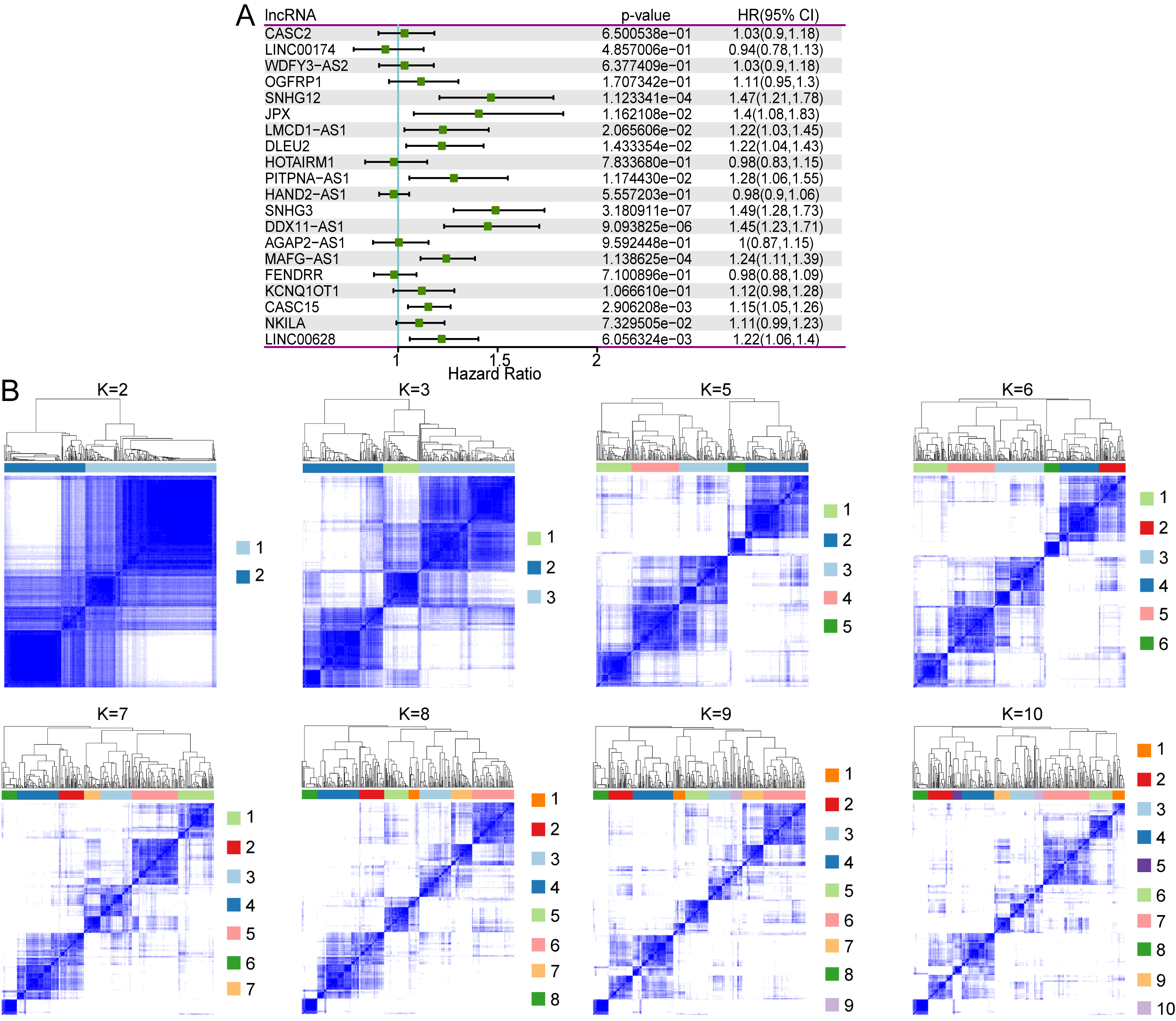

Supplement: Supplementary file 1 — Supplementary Figure 1. [file 41598_2022_13013_MOESM1_ESM.tif]

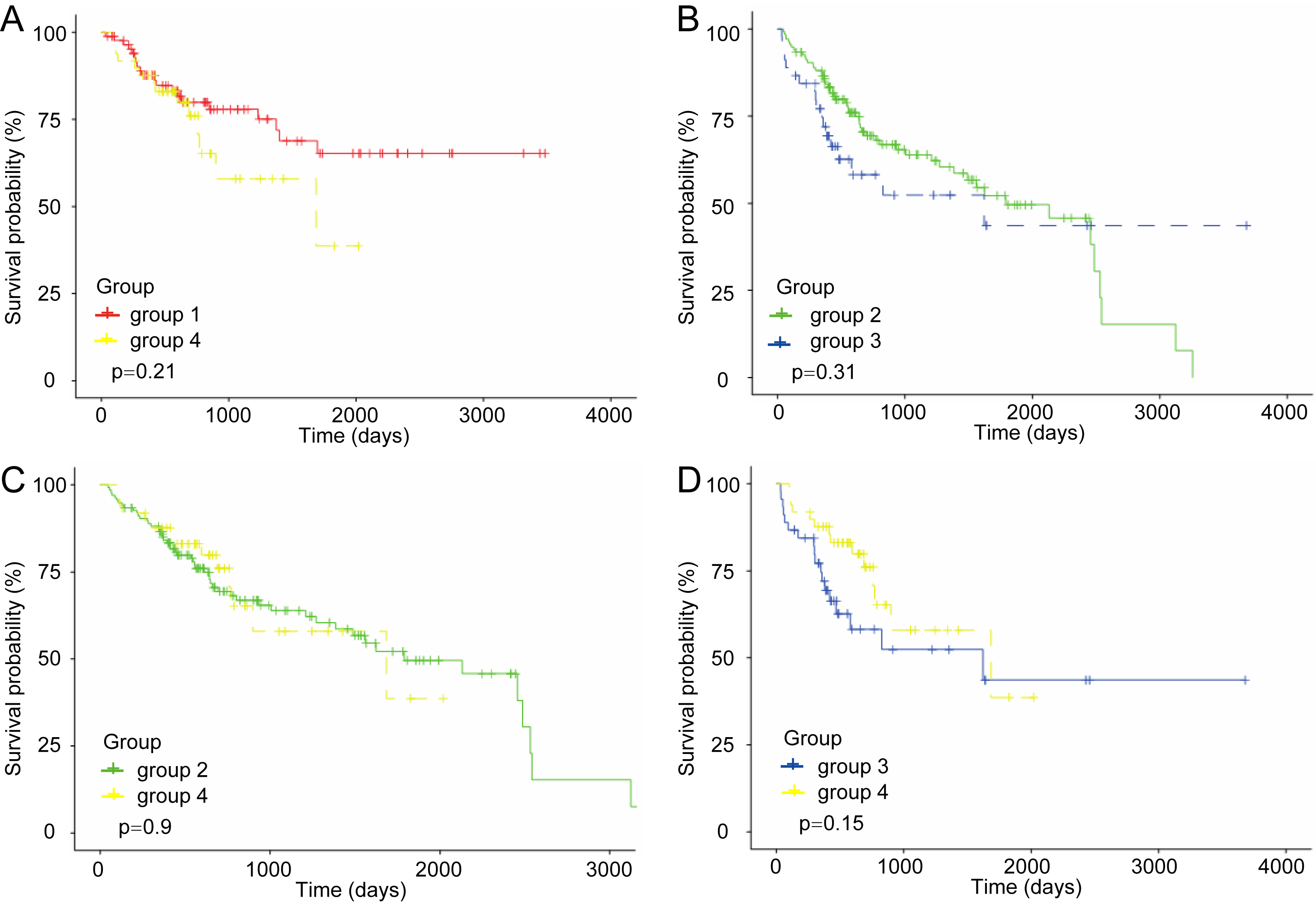

Supplement: Supplementary file 2 — Supplementary Figure 2. [file 41598_2022_13013_MOESM2_ESM.tif]

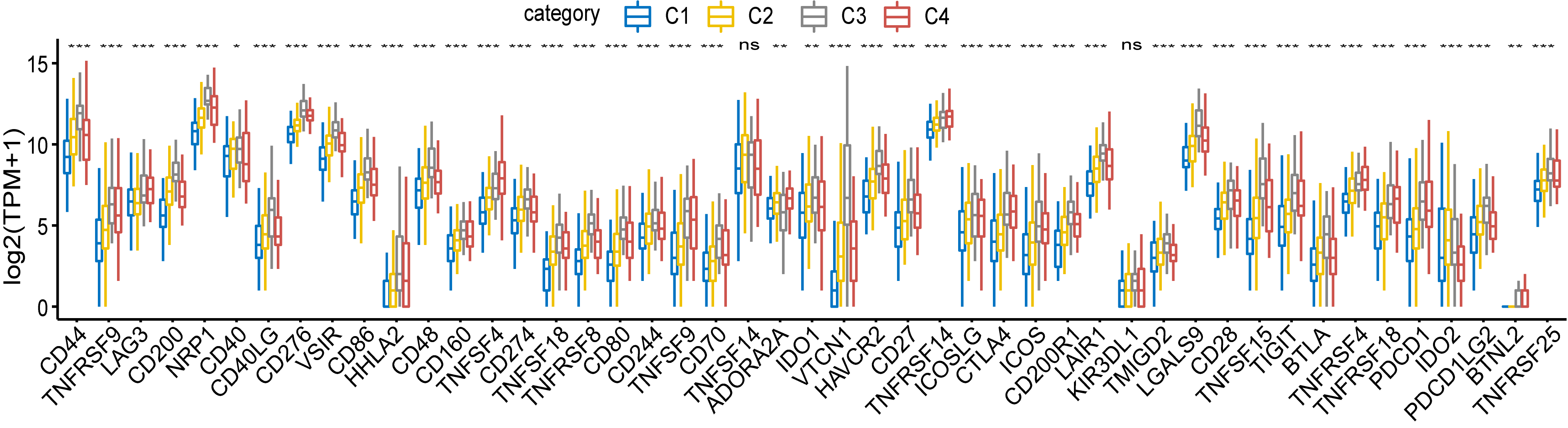

Supplement: Supplementary file 3 — Supplementary Figure 3. [file 41598_2022_13013_MOESM3_ESM.tif]

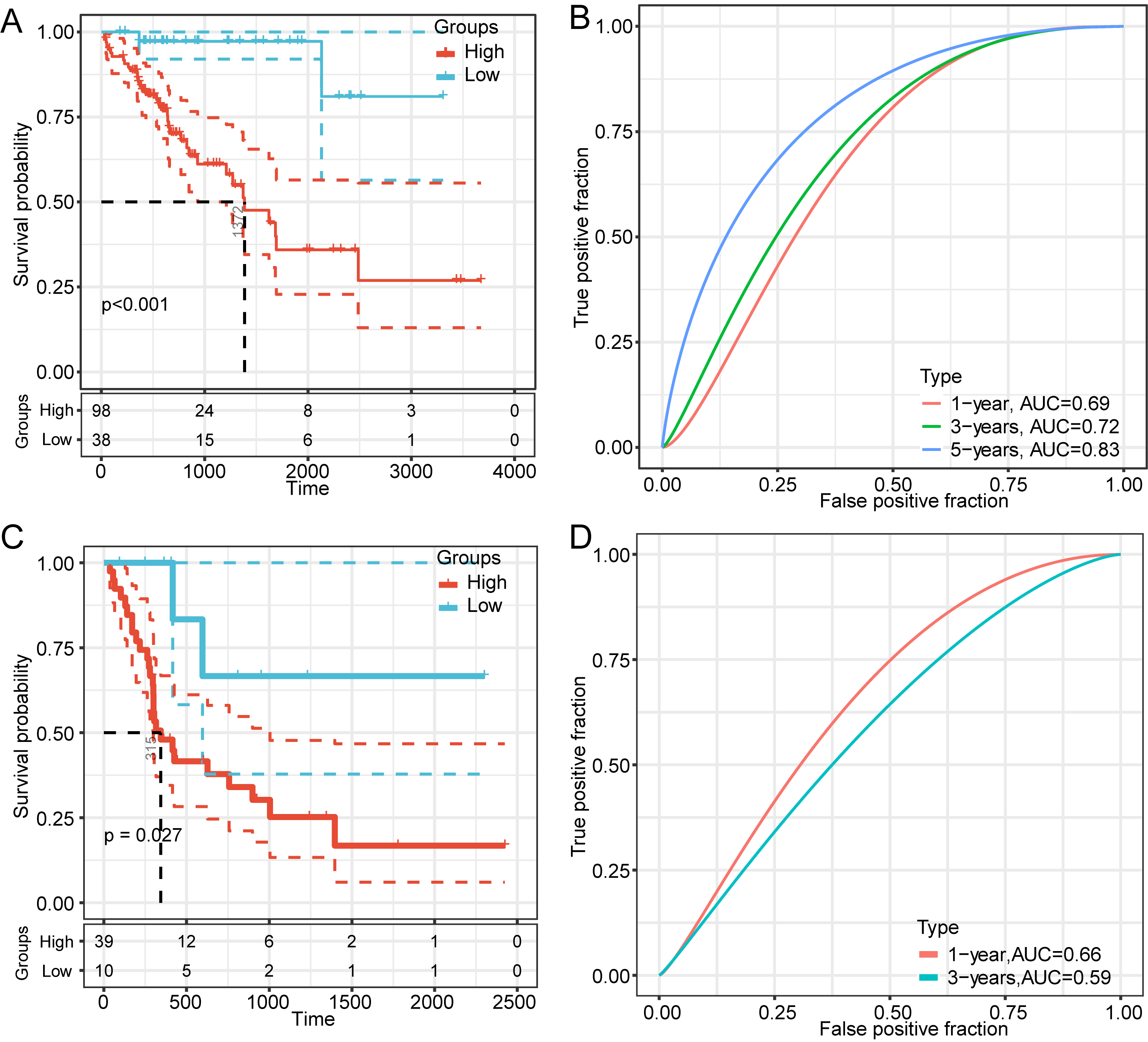

Supplement: Supplementary file 4 — Supplementary Figure 4. [file 41598_2022_13013_MOESM4_ESM.tif]
