# Supplementary material for: Molecular characteristics of novel immune subtypes of HCC based on lncRNAs related to immune disorders
Source: Sci Rep. 2022 May 26;12:8905. doi: 10.1038/s41598-022-13013-7 (PMC9135727; doi:10.1038/s41598-022-13013-7)
Supplement: Supplementary file 5 — Supplementary Legends. [file 41598_2022_13013_MOESM5_ESM.docx]

**Figure legends**

**Supplementary Figure 1** A, The prognostic value of 20 immune-related lncRNAs in HCC. B, Consensus matrix when k = 2–10.

**Supplementary Figure 2** Cross-analysis of KM curves between subtypes.

**Supplementary Figure 3** Differential expression of immune checkpoint genes between groups.

**Supplementary Figure 4** Performance of prognostic gene signatures in group 2 and group 4. A, The prognostic model we identified had significant prognostic significance between the high- and low-risk groups classified in group 2. B, ROC analysis of prognostic model in group 2. C, The prognostic model we identified had significant prognostic significance between the high- and low-risk groups classified in group 4, D，ROC analysis of prognostic model in group 4.
