# Supplementary material for: Molecular characteristics of novel immune subtypes of HCC based on lncRNAs related to immune disorders
Source: Sci Rep. 2022 May 26;12:8905. doi: 10.1038/s41598-022-13013-7 (PMC9135727; doi:10.1038/s41598-022-13013-7)
Supplement: Supplementary file 12 — Supplementary Legends. [file 41598_2022_13013_MOESM12_ESM.docx]

**Supplementary Table Legend**

**Supplementary Table 1** Immune-related pathways in ssGSEA were used to screen immune-related lncRNAs.

**Supplementary Table 2** There were 151 lncRNAs with lncRES>0.995, which were considered to be lncRNAs associated with immune.

**Supplementary Table 3** 634 immune disorder-related lncRNAs identified based on the Immune Cell Abundance Identifier (ImmuCellAI) method.

**Supplementary Table 4** The intersection of the above two immune-related lncRNAs was assessed and 20 immune-related lncRNAs were identified in HCC.

**Supplementary Table 5** List of differentially expressed genes between Group 1 and Group 3.

**Supplementary Table 6** List of differentially expressed genes with prognostic value between Group 1 and Group 3.
